# The Effect of Biochar Addition on Thermal Stability and Decomposition Mechanism of Poly(butylene succinate) Bionanocomposites

**DOI:** 10.3390/molecules28145330

**Published:** 2023-07-11

**Authors:** Katerina Papadopoulou, Evangelia Tarani, Nina Maria Ainali, Konstantinos Chrissafis, Christian Wurzer, Ondřej Mašek, Dimitrios N. Bikiaris

**Affiliations:** 1 Laboratory of Polymer Chemistry and Technology, Department of Chemistry, Aristotle University of Thessaloniki, GR-54124 Thessaloniki, Greece; katerina_1991papa@hotmail.com (K.P.); ainali.nina@gmail.com (N.M.A.); 2 Laboratory of Advanced Materials and Devices, Department of Physics, Aristotle University of Thessaloniki, GR-54124 Thessaloniki, Greece; etarani@physics.auth.gr (E.T.); hrisafis@physics.auth.gr (K.C.); 3 UK Biochar Research Centre, School of GeoSciences, University of Edinburgh, Alexander Crum Brown Road, Edinburgh EH9 3FF, UK; c.wurzer@ed.ac.uk (C.W.); ondrej.masek@ed.ac.uk (O.M.)

**Keywords:** poly(butylene succinate), biochar, nanocomposites, thermal degradation, decomposition mechanism

## Abstract

In the present study, poly(butylene succinate) (PBSu) and its bionanocomposites containing 1, 2.5, and 5 wt.% biochar (MSP700) were prepared via in situ melt polycondensation in order to investigate the thermal stability and decomposition mechanism of the materials. X-ray photoelectron spectroscopy (XPS) measurements were carried out to analyze the surface area of a biochar sample and PBSu/biochar nanocomposites. From XPS, it was found that only physical interactions were taking place between PBSu matrix and biochar nanoadditive. Thermal stability, decomposition kinetics, and the decomposition mechanism of the pristine PBSu and PBSu/biochar nanocomposites were thoroughly studied by thermogravimetric analysis (TGA) and pyrolysis–gas chromatography/mass spectrometry (Py−GC/MS). TGA thermograms depicted that all materials had high thermal stability, since their decomposition started at around 300 °C. However, results indicated a slight reduction in the thermal stability of the PBSu biochar nanocomposites because of the potential catalytic impact of biochar. Py−GC/MS analysis was employed to examine, in more detail, the thermal degradation mechanism of PBSu nanocomposites filled with biochar. From the decomposition products identified by Py−GC/MS after pyrolysis at 450 °C, it was found that the decomposition pathway of the PBSu/biochar nanocomposites took place mainly via β-hydrogen bond scission, which is similar to that which took place for neat PBSu. However, at higher biochar content (5 wt.%), some localized differences in the intensity of the peaks of some specific thermal degradation products could be recognized, indicating that α-hydrogen bond scission was also taking place. A study of the thermal stability and decomposition pathway of PBSu/biochar bionanocomposites is crucial to examine if the new materials fulfill the requirements for further investigation for mulch films in agriculture or in electronics as possible applications.

## 1. Introduction

In the last few decades, biobased and biodegradable polymers have attracted considerable attention because of the serious problems of environmental pollution, global warming, and fossil-fuel depletion. In response to these environmental issues, “biobased polymers” and/or “green polymers” are used as alternative and ecofriendly materials in a high number of applications [1].

PBSu is one of the most interesting biobased and biodegradable aliphatic polyesters; accordingly, it has been intensively studied and commercially produced. PBSu was used for the first time in the 1990s and has since been used in the industry [2]. Its prominent place among the most promising biobased and biodegradable aliphatic thermoplastic polyesters is due to its excellent characteristics, which are comparable to those of worldwide used fossil-derived polymers. Regarding its properties, it has been investigated for new possible applications as a novel material for ecological purposes in agriculture [3,4,5,6,7]. It is synthesized through a polycondensation reaction between succinic acid and 1,4-butanodiol, which can be produced via the fermentation method; thus, it can be characterized as a biobased monomer [8,9]. One reason why PBSu has gained commercial availability is that it presents some interesting properties, such as controllable biodegradability, good physical properties, melt processability, and chemical resistance. However, there are disadvantages related to the use of PBSu, since it presents a high production cost and poor thermal and mechanical properties [2,4,5,10]. The use of additives to produce nanocomposites can help to improve its drawbacks, as well as reduce costs; as such, nanocomposites have been extensively investigated [11,12,13,14,15,16,17].

Due to the increasing interest in ecofriendly and sustainable materials, biochar has been intensively studied as a promising filler and reinforcement in polymers [18]. Biochar is a byproduct of biomass pyrolysis and, thus, can be characterized as a biobased filler. The properties of biochar vary according to the derived biomass and the pyrolysis conditions [19,20]. The growing popularity of biochar is due to its dual nature. It can not only serve as a filler due to the charcoal powder of biochar, but also be used as a reinforcement agent by improving the thermomechanical properties [21,22,23,24,25]. Some of its beneficial characteristics are high carbon content [26], high chemical and thermal stability [22,25], cost-competitiveness, and abundance of material. Furthermore, the thermal stability of biochar is a fundamental characteristic that makes it a desirable material for various applications in different fields. Thus, the incorporation of biochar in the polymer matrix is possible for potential applications (e.g., in agriculture as mulch films and in electronics).

Many studies on the thermal degradation kinetics with several types of fillers such as carbon nanotubes (CNTs), graphene nanoplatelets (GNs), and multiwalled carbon nanotubes (MWCNTs) have been carried out. From the study of Song et al. [27], it seems that the addition of CNTs improved the heat resistance of nanocomposites. The results of Platnieks et al. [6] showed that PBSu/GNs nanocomposites had slightly improved thermal stability. Furthermore, the thermal degradation kinetics study by Ray et al. [28] proved that the thermal stability of PBSu/MWCNTs nanocomposites is higher compared to that of pristine PBSu.

In our previous work, PBSu/biochar bionanocomposites were prepared using in situ polymerization and characterized as a function of thermal conductivity, diffusivity and crystallization kinetics, mechanical properties, and molecular mobility [29,30]. Transmission electron microscopy (TEM) revealed that biochar is dispersed on a nanoscale in the PBSu matrix. Thus, our work confirmed that biochar can act as an effective reinforcing [29,30] and crystallization agent [29,30]. In addition to the mechanical properties, the thermal stability of a polymer is very important for processing and applications. It is well known that the incorporation of nanofillers in pristine polymers can enhance the thermal stability of nanocomposites and can play a crucial role in the decomposition mechanism. However, to date, there is no published work in the literature concerning a comparative study of the thermal degradation of PBSu nanocomposites with biochar. Therefore, the aim of the present work is to investigate the thermal stability and decomposition mechanism of the PBSu/biochar nanocomposites before their application in different fields.

## 2. Results and Discussion

### 2.1. X-ray Photoelectron Spectroscopy (XPS)

**Figure 1:**
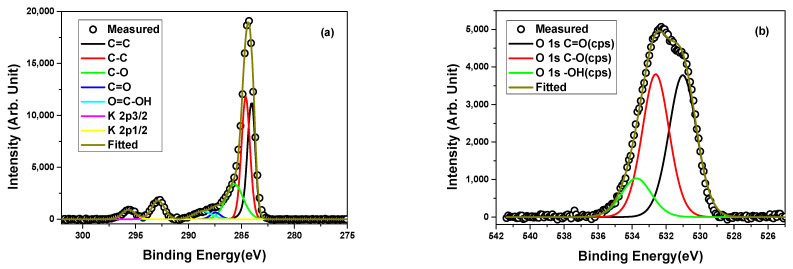
High-resolution XPS spectra of (**a**) C 1s and (**b**) O 1s core levels and peak deconvolution for biochar.

As determined in our previous work by TEM, biochar is dispersed in the PBSu matrix at a nanoscale, acting as an effective reinforcing agent [29,30], whereby it improves the mechanical properties. This dispersion could be a result of the evolved interactions taking place between biochar and the PBSu matrix, which could also affect the thermal decomposition of nanocomposites. XPS is a powerful technique used to investigate the chemical composition and electronic states of surfaces; for this reason, it was used in the present work. In recent years, XPS has been increasingly applied to study biochar, a carbon-rich material produced by the pyrolysis of biomass. XPS measurements were carried out to analyze the surface area of a biochar sample. Figure 1 shows the high-resolution XPS spectra of C 1s and O 1s core levels, as well as the peak deconvolution for the Miscanthus biochar sample (MSP700) used in this study. The photoelectrons released from the C 1s core level and the O 1s core level were responsible for the peaks seen at the binding energies of ~284 eV and ~532 eV, respectively. In addition to C and O, mineral elements (Si, P, and Ca) were also determined in very small amounts on the biochar surface by XPS analysis. As shown in Table 1, the C 1s high-resolution spectra of biochar could be fitted with seven peaks. The peak at 284 eV was ascribed to the sp^2^ hybridized graphitic structure. The peak at 284.6 eV was assigned to the sp^3^ hybridized orbital of diamond-like carbon. The peaks with higher binding energies situated at 285.7, 287.4, and 288.7 eV corresponded to C–O (epoxy groups), C=O (carbonyl), and O=C–OH (carboxylic acid groups), respectively. This is in accordance with the literature results [31,32]. Furthermore, the C K-edge absorption spectrum was acquired, providing a direct measurement of the vacant orbitals of the carbon atoms. The spectrum showed several near-edge absorption features that provide insight into the electronic properties of the carbon atoms. One interesting feature of the spectrum was a small peak at 285 eV that could not be fitted. This peak was due to the electronic transition from the C 1s core level to the π* orbital, which is a measure of the presence of unsaturated C–C bonds [33]. The presence of unsaturated C–C bonds is a crucial aspect of many carbon-based materials, including graphene and carbon nanotubes. The unoccupied π* orbital is also important in understanding the reactivity of carbon-based materials. It can participate in chemical reactions and influence the behavior of molecules and ions in contact with the material.

The higher peak intensities at ~531.0 eV, ~532.6 eV, and ~533.7 eV in the O 1s spectrum confirmed the presence of oxygen atoms in the form of C=O, C–O, and –OH, respectively (Figure 1b). The spectra of biochar showed a small amount of oxygen, indicating that there were some oxidized contaminants in the starting materials. The oxygen-containing functional groups found on the surface of biochar were responsible for these peaks. Epoxy groups, which are frequently present in biochar made from biomass with a high lignin concentration, were responsible for the C–O peak. The C=O peak was attributed to carbonyl groups formed during the pyrolysis process. The O=C–OH peak was due to carboxylic acid groups, which could have been formed by the oxidation of carbonaceous materials during the production of biochar. The presence of functional groups such as epoxy, carbonyl, and carboxylic acid can influence the reactivity and adsorption capacity of biochar, which can affect its properties and performance.

**Figure 2:**
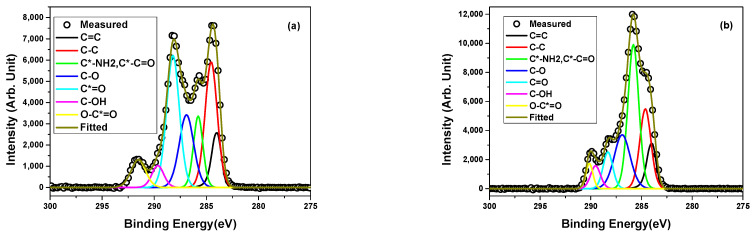
High-resolution XPS spectra of C 1s core level and peak deconvolution for (**a**) neat PBSu and (**b**) PBSu/biochar 5 wt.% nanocomposite.

XPS was used to conduct further characterization of the PBSu/biochar nanocomposites in the study. Through the application of XPS, the chemical bonding state and information of various atoms including C, O, and N were analyzed. Additionally, the impact of incorporating biochar on the binding energy of atomic chemical bonds was explored, and the interaction between molecules was studied by examining the effect of filler addition on the binding energy of atomic chemical bonds. The high-resolution spectra of the C 1s core levels were deconvoluted to separate the various bond contributions in Figure 2. Table 2 shows the chemical bonding energies of nanocomposites (C 1s) of neat PBSu and PBSu/biochar 5 wt.%. The decomposition of the C 1s peak of neat PBSu demonstrated the existence of seven components: sp^2^ carbon(C=C), aliphatic carbon (C–C (or C–H)), C*–NH_2_/C*–C=O, C*–O, C*=O, C*–OH, and O–C*=O. The asterisk denotes the atom targeted by the measurement. It is well known that the binding energy of C*–NH_2_ is 285.5–285.8 eV, while that of C*–C=O is 285.2–285.6 eV [34]. Because of the binding energy overlap, these bonds are considered to be one component.

The PBSu/biochar 5 wt.% nanocomposite’s C 1s peak was also deconvoluted into seven subpeaks. In detail, the C 1s core level analysis for PBSu/biochar 5 wt.% confirmed the presence of C=C, C–C/C–H, C–OH, C–O, C=O, C–OH, and O–C=O bonds. However, it was found that the atomic contents of the various elements in neat PBSu were 27.30% O 1s, 71.51% C 1s, and 1.19% N 1s, while PBSu/biochar 5 wt.% contained 27.61% O 1s, 71.91% C 1s, and 0.48% N 1s. The carbon content on the surface of the PBSu/biochar 5 wt.% nanocomposite was a little bit higher than that of neat PBSu. Because of this, it was found that the PBSu/biochar 5 wt.% mixture had a slightly higher E_binding_ for O 1s and C 1s than neat PBSu. The increase in the carbon content on the surface of the nanocomposite suggests that biochar is effectively incorporated into the polymer matrix. The high surface area of biochar nanoparticles may provide more opportunities for physical interactions with the surrounding polymer matrix. Neratzaki et al. [35] demonstrated that there were no chemical interactions or covalent bonding between SrHAp nanorods and the PBSu matrix. However, small differences were observed in the oxygen region, suggesting the presence of physical interactions between PBSu and the nanoparticles. Jacquel et al. [36] conducted a study on in situ synthesized PBSu/silica nanocomposites, exploring the relationship between their structure and properties, and they observed the presence of physical interactions between PBSu and silica. Zhang et al. [37] found that the positions and intensities of characteristic peaks remained largely unchanged upon the introduction of nanoparticles, indicating the formation of weak physical interactions rather than chemical interactions between the nanoparticles and the poly(butylene succinate-co-terephthalate) matrix. This is in accordance with our previous work [30], in which the IR spectra retained most of the band of PBSu. Additionally, the intensity of O–C=O chemical bonding in the PBSu/biochar 5 wt.% nanocomposite shifted toward lower binding energy, indicating the occurrence of an interaction between the ester group and other groups [38], most probably from the biochar. 

**Figure 3:**
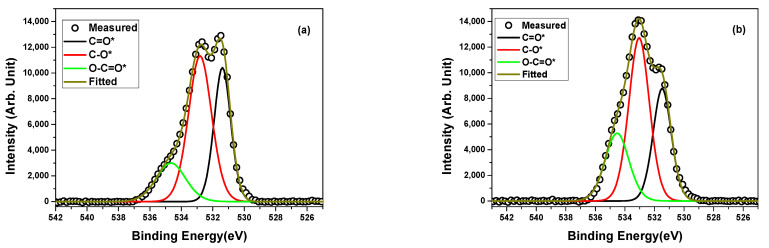
High-resolution XPS spectra of O 1s core level and peak deconvolution for (**a**) neat PBSu and (**b**) PBSu/biochar 5 wt.% nanocomposite.

To understand how the PBSu/biochar nanocomposite works, it was important to look into the surface properties of the O 1s and N 1s XPS spectra. It is worth mentioning that the carbon spectrum showed seven different chemical states. However, oxygen and nitrogen had three and two different chemical states, respectively. There was a difference observed in the binding energy (E_binding_) of the three elements between neat PBSu and the PBSu/biochar 5 wt.% nanocomposite. In detail, E_binding_ values for O 1s, C 1s, and N 1s were found to be 531.6 eV, 284.4 eV, and 399.4 eV for neat PBSu and 532 eV, 285.9 eV, and 399.3 eV for the PBSu/biochar 5 wt.% nanocomposite, respectively. Figure 3 displays the deconvoluted spectra, and Table 3 lists the results of the quantitative study. The O 1s core-level analysis for neat PBSu and the PBSu/biochar 5 wt.% nanocomposite showed peaks corresponding to different oxygen-containing functional groups. For example, the peak at ~531.4 eV was attributed to C=O bonds, while the peak at ~532.8 eV was attributed to C–O bonds. The peak at ~534.7 eV was assigned to O–C=O bonds. These groups are also shown in the spectrum of C 1s in Figure 2; the peak of the carbonyl group was located at ~288.2 eV, the peak of C–O was found near ~286.9 eV, and the corresponding peak of O–C=O was located at ~290.5 eV. The O/C ratio measured from the PBSu surface changed from 38.2% to 38.4% after the incorporation of biochar into the polymer matrix.

**Figure 4:**
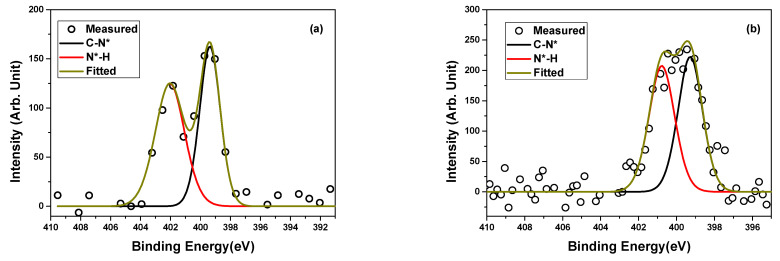
High-resolution XPS spectra of N 1s core level and peak deconvolution for (**a**) neat PBSu and (**b**) PBSu/biochar 5 wt.% nanocomposite.

XPS was also used to investigate the N 1s spectra of neat PBSu and the PBSu/biochar 5 wt.% nanocomposite. The C–N and N–H bonds had corresponding peak components at ~399.4 and ~400.2 eV in the N 1s spectra of neat PBSu and the PBSu/biochar 5 wt.% nanocomposite, respectively, in Figure 4. The C–N peak at ~399.4 eV could be attributed to the nitrogen atoms in the PBSu polymer backbone, which are bonded to carbon atoms through a single bond. This bond is typically associated with primary amine and/or imine functional groups in the PBSu polymer. The N–H peak at ~400.2 eV could be attributed to the nitrogen atoms in the PBSu polymer chains that are bonded to hydrogen atoms. This peak was indicative of the presence of amide and/or amine functional groups in the PBSu polymer. The presence of N–H and N–C bonds originates from the natural chemical structure of the polymer and the reactions that occur during its synthesis. These functional groups are commonly found in polymeric materials, and they contribute to the overall chemical composition of the polymer. The N 1s spectra of the composite were not considerably changed by the addition of biochar to PBSu. Both peaks were still present and exhibited similar peak positions in the spectra. Interestingly, the percentage area under the peaks changed in the spectrum of the nanocomposite compared to that of neat PBSu. The intensity of the C–N bond in the PBSu/biochar 5 wt.% nanocomposite was higher than that of neat PBSu, indicating an increase in the nitrogen content of the nanocomposite.

### 2.2. Thermal Degradation Study of PBSu/Biochar Nanocomposites

**Figure 5:**
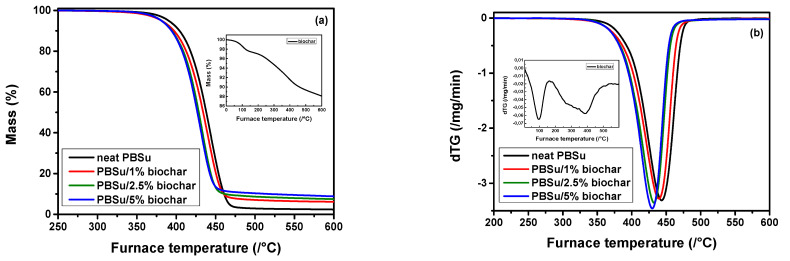
(**a**) TGA thermograms and (**b**) dTG curves of PBSu/biochar nanocomposites at a heating rate of 20 °C/min under a nitrogen atmosphere.

Using TGA, the thermal degradation of PBSu nanocomposites was investigated. At a heating rate of 20 °C/min in a nitrogen environment, Figure 5 shows the TGA thermograms and dTG curves of neat PBSu, biochar (shown inset), and PBSu/biochar nanocomposites. According to the TGA results of the biochar sample, the first degradation stage up to 150 °C was related to water loss, while the subsequent weight loss could be largely attributed to the decomposition of organic matter [39]. The results showed that biochar exhibits a stable behavior, with a minimal mass loss of <15% up to 600 °C. This outcome was expected, given that biochar is already subjected to thermal degradation during the pyro-gasification process [18] The thermal profiles observed for biochar are typical of a pyrolyzed carbonaceous material derived from agricultural and forestry waste. The slow rate of mass loss observed in the thermograms was mainly attributed to the presence of refractory organic matter, degraded lignin, and strong C–C covalent bonds that are resistant to degradation [18]. The C 1s high-resolution spectra from XPS analysis indicated peaks corresponding to graphitic and diamond-like carbon structures, as well as various functional groups such as epoxy, carbonyl, and carboxylic acid groups. Overall, the thermal stability of biochar is an essential property that makes it an attractive material for various applications.

From the TGA curves, it can be seen that neat PBSu and the PBSu/biochar nanocomposites presented good thermostability with no significant mass loss up to 300 °C. Table 4 shows the temperatures at which the PBSu nanocomposites underwent 0.5%, 2.5%, and 5% mass loss. The results indicate a reduction in the thermal stability of the PBSu biochar nanocomposites. With an increase in filler content, both the onset and the maximum thermal degradation temperatures of the PBSu nanocomposites decreased. This was due to the fact that biochar had a lower onset and maximum thermal degradation temperature compared to neat PBSu. The physical interaction observed in XPS analysis can influence the stability and integrity of the polymer matrix during thermal degradation. Additionally, lower-molecular-weight polymers tend to have lower thermal stability because their shorter chain lengths allow for more rapid molecular mobility and easier access to the polymer backbone by degrading agents. In our previous work [29], the molecular weight was found to slightly decrease with increasing biochar content. In the study conducted by Mamun et al. [40], it was reported that the addition of low-molecular-weight polystyrene (PS) to the PCL/PS blend resulted in a decrease in thermal stability. Conversely, the inclusion of high-molecular-weight PS was found to improve the thermal stability of the blend. The residue content of the PBSu nanocomposites at 600 °C was found to increase with increasing the biochar content due to the accumulation of a higher char amount able to persist at high temperatures [41,42].

The highest decomposition temperatures for PBSu nanocomposites with 1, 2.5, and 5 wt.% biochar fillers occurred at 439.4 °C, 431.9 °C, and 429.7 °C, respectively, according to the dTG curves, indicating that these nanocomposites decomposed faster than neat PBSu. This small drop in breakdown temperature is evidence of the biochar filler having a potent catalytic impact, greatly lowering the thermal stability of PBSu nanocomposites. Biochar, being a pyrolyzed carbonaceous material, contains organic matter that decomposes at lower temperatures.

The degree of conversion (α) and kinetic parameters were calculated in order to describe the degradation mechanism of neat PBSu and the PBSu/biochar 5 wt.% nanocomposite. Thus, the isoconversional procedures and the model fitting methods were applied. Under a nitrogen atmosphere, the mass curves were measured at different heating rates of 5, 10, 15, and 20 °C/min [43]. The generic equation for solid-state reactions can be used to define the reaction rate:

(1) $$\frac{d\alpha }{dt}=k\left(T\right)f\left(\alpha \right),$$

where α is the degree of conversion, f(α) is the reaction model, and k(T) is the reaction rate constant. The degree of conversion α is the ratio of actual mass loss at a particular temperature Δm to total mass loss Δm_tot_ that happens after the degrading process is complete:

(2) $$\alpha =\frac{m_{0}-m}{m_{0}-m_{f}}=\frac{\Delta m}{\Delta m_{tot}}.$$

The algebraic expression known as the kinetic model, or f(α), characterizes the solid-state reaction’s kinetics. The Arrhenius equation, which states that $k\left(T\right)={\mathrm{Ae}}^{-E/\mathrm{RT}}$, describes how temperature affects reaction rate, where E is the apparent activation energy (kJ/mol), R is the gas constant (8.314 J/mol·K), A is the pre-exponential factor (s^−1^), and T is the absolute temperature (K). 

Differential and integral procedures make up the isoconversional techniques. The differential isoconversional method of Friedman [44], the integral isoconversional method of Vyazovkin [45], and the integral isoconversional method of Ozawa, Flynn, and Wall (OFW) [46] are the most frequently utilized methods. The differential isoconversional method of Friedman can be expressed as

(3) $$ln\left[\beta_{i}{\left(\frac{d\alpha }{dt}\right)}_{\alpha ,i}\right]=\ln\left[f\left(\alpha \right)A_{\alpha }\right]-\frac{E_{\alpha }}{RT_{\alpha ,i}},$$

where β is the heating rate, and A is the pre-exponential factor. It is necessary to determine the slope of the straight lines in the plot of ln[β_i_(dα/dt)_α,i_] vs. 1/T_α,i_ in order to derive the activation energy E values at a constant conversion function. Differential approaches have the benefit of being applicable to any temperature program and requiring no approximations. However, compared to integral ones, they may exhibit numerical instability due to a determination limit on the baseline. 

Vyazovkin suggested an isoconversional nonlinear approach to determine the E_α_:

(4) $$\Phi \left(E_{\alpha }\right)=\overset{n}{\underset{i=1}{\sum }}\overset{n}{\underset{j\neq 1}{\sum }}\frac{J\left[E_{\alpha },{Τ}_{i}\left(t_{\alpha }\right)\right]}{J\left[E_{\alpha },{Τ}_{j}\left(t_{\alpha }\right)\right]}\;,$$

where *i* and *j* represent a series of trials carried out at various heating rates, n represents the total number of experiments, and *J* is assessed throughout over small intervals of E_α_ variation:

(5) $$J\left[E_{\alpha },{Τ}_{i}\left(t_{\alpha }\right)\right]=\overset{t_{\alpha }}{\underset{t_{\alpha -\Delta \alpha }}{\int }}exp\left[\frac{-E_{\alpha }}{RT_{i}\left(t\right)}\right]dt.$$

E_α_ is calculated using Equation (4), which is the value that minimizes the function Φ(E_α_). For each i-th temperature program, an exact interpolation using a Lagrangian algorithm determines the time t_α,i_ and temperature T_α,i_ of chosen values.

The equation proposed by Ozawa, Flynn, and Wall is as follows:

(6) $$\ln\left(\beta_{i}\right)=Const-1.052\left(\frac{E_{\alpha }}{RT_{\alpha }}\right),$$

where β is the heating rate given by β=dT/dt=const. The various heating rates that were applied to the experimental data are expressed by the index i. The slope of the ln(β_i_) vs. 1/T_α_ plots can be used to determine the activation energy E values.

**Figure 6:**
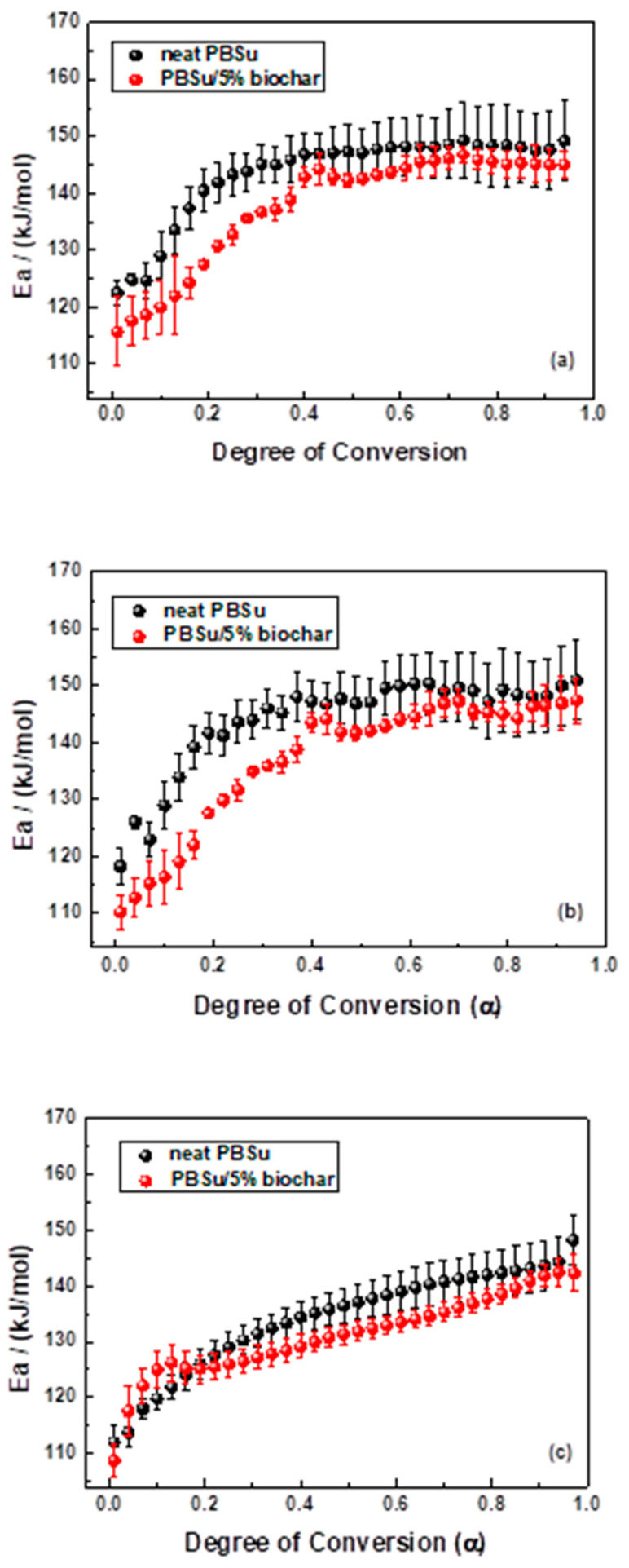
The relationship between the activation energy (E_α_) and the degree of conversion (α) for thermal degradation of neat PΒSu and PΒSu/biochar 5 wt.% nanocomposite, as determined by the (**a**) Friedman method, (**b**) Vyazovkin analysis, and (**c**) Ozawa–Flynn–Wall analysis.

The activation energies were calculated using the three isoconversional methods: the differential isoconversional approach of Friedman, the integral isoconversional method of Vyazovkin, and the integral isoconversional method of OFW [44,45,47]. Using the aforementioned isoconversional methods, Figure 6 plots the E_α_ values of neat PBSu and the PBSu/5% biochar nanocomposite against the degree of conversion. The E_α_ mean values calculated by the Friedman technique and the Vyazovkin method from the TGA curves were found to be in good agreement. The E-dependency in all cases indicates that the thermal degradation mechanism was complicated, and that the start and final stages of the degradation kinetics were controlled by various mechanisms. While they decreased for higher values, the E_α_ values of neat PBSu and PBSu/biochar 5 wt.% nanocomposite showed an increase in the first area. The E_α_ mean values of neat PBSu for 0.2 were determined to be 129.6, 127.9, and 118.3 kJ/mol, respectively, according to the Friedman technique, Vyazovkin analysis, and OFW method, while the values for >0.2 were 146.9, 148.0, and 138.3 kJ/mol, respectively. Additionally, the E_α_ values of PBSu/biochar 5 wt.% nanocomposite (α < 0.1) were 124.0. 115.7, and 118.0 kJ/mol for Friedman method, Vyazovkin analysis, and OFW method, respectively, and 140.3, 140.3, and 133.3 kJ/mol for α > 0.1. The thermal decomposition of the PBSu/biochar 5 wt.% nanocomposite required less activation energy than that of neat PBSu.

**Figure 7:**
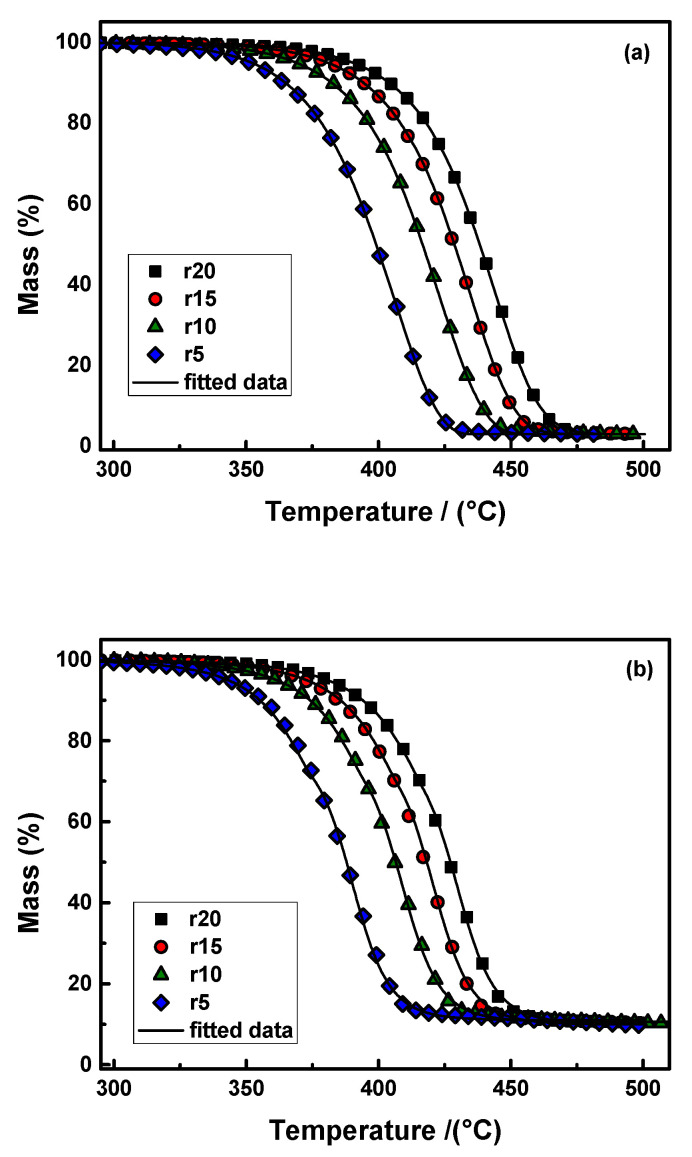
The mass (%) curves and the corresponding fitting curves with the combination of Cn–Cn reaction models (continuous black lines) of (**a**) neat PBSu and (**b**) PBSu/biochar 5 wt.% for heating rates of 5, 10, 15, and 20 °C/min in a nitrogen environment.

By comparing experimental (four heating rates) and theoretical data, multivariate nonlinear regression or model-fitting methods were used to identify the mechanism of degradation and the kinetic triplet (E, A, f(α)) of each reaction. For the fitting of the experimental data at various heating rates, 16 distinct reaction models were used. First, it was presumed that a single-step reaction process takes place, which correlates to the significant mass loss. Two or more mechanisms were merged if the fitting to the experimental data did not produce satisfactory results. In our previous work [48], we found that the mechanism of autocatalysis n-order (Cn) is the form of the conversion function that best describes neat PBSu:

(7) $$f\left(\alpha \right)={\left(1-\alpha \right)}^{n}\left(1+K_{cat}X\right),$$

where X represents the degree of conversion of the autocatalytic reactions, and K_cat_ is the autocatalysis rate constant. The autocatalytic reaction says that when polymer fragments broken down by heat (reaction products) attack the polymer’s unreacted parts, they break the chemical bonds and speed up the breakdown process. The combination of two consecutive Cn–Cn reaction models fit the experimental results of neat PBSu and PBSu/biochar 5 wt.% nanocomposite well, as shown in Figure 7. The experimental data were used to fit the two consecutive Cn–Cn reaction models significantly better than the single reaction model. This is consistent with the findings in the literature [48,49]. For neat PBSu and PBSu/biochar 5 wt.% nanocomposite, the correlation coefficient (R^2^) values were found to be 0.99997 and 0.99990, respectively. Hence, the degradation of both studied samples took place in two stages: the first corresponding to a very small mass loss, and the second at elevated temperatures being the main degradation stage. The first decomposition step was due to oligomer degradation [50]. Table 5 shows a summary of the calculated parameters (activation energy, pre-exponential factor, and reaction order) for the tested models. The E_α_ values from the two-step mechanism model and those from the isoconversional methods agreed well. According to the estimated values, adding biochar filler to the PBSu matrix lowered the E_α_ values and greatly increased the decomposition rate constant. The lower activation energy required for the thermal decomposition of the PBSu/biochar 5 wt.% nanocomposite compared to neat PBSu makes it an interesting candidate for novel materials with ecological applications in agriculture. This property suggests that the nanocomposite can undergo thermal degradation and decomposition at lower temperatures with higher efficiency. Furthermore, the pre-exponential part of the PBSu/biochar 5 wt.% nanocomposite had lower values in both stages compared to neat PBSu after the E values were calculated. Because the rate constant of the PBSu/5% biochar mixture was much higher than that of neat PBSu, this sped up the thermal degradation. Thus, the observed decrease in activation energy and higher decomposition rate constant in the PBSu/biochar nanocomposite suggest that the biochar filler influenced the degradation mechanism. The interactions between the polymer matrix and biochar may have led to enhanced degradation pathways and increased reactivity, resulting in faster degradation rates.

### 2.3. Pyrolysis−Gas Chromatography/Mass Spectrometry Analysis (Py−GC/MS)

The thermal stability of a polymer constitutes a very important property which initially defines the synthetic procedure and subsequently determines the polymer applications. In this framework, the thermal degradation pathway of polymers, especially polyesters, has been outlined as an investigation of great importance, since these materials hold an outstanding role in the plastics market, along with other industrial polymeric materials [51,52]. Herein, to completely optimize the thermal stability profile of polymeric structures, the investigation of thermal decomposition pathways was considered of crucial importance. Although widely utilized thermal analytical instrumentations, such as thermogravimetric analysis (TGA), deliver important information about the thermal stability and the kinetics of thermally induced polymer decomposition, a Py−GC/MS analysis can create and provide valuable data about the specific pathway of the thermal degradation of polymeric structures.

Poly(n-alkylene succinate)s constitute a class of highly promising polyesters, mainly due to their biodegradable nature. Concerning the relative polyester with the methylene group numbers in the diol segment n = 4, poly(butylene succinate) (PBSu) is an aliphatic polyester whose thermal degradation pathway was previously evaluated by our team [48,50]. Additionally, since the synthesis and the examination of the physicochemical properties of the PBSu/biochar nanocomposites were conducted in our recently reported work [29,30], in the framework of the present study, the effect on the thermal decomposition profile of the new materials after the incorporation of biochar into the succinic counterpart was evaluated. According to the available data, the high volatile content of untreated biomass, was considerably decreased throughout carbonization, rendering biochar a more thermally stable material. Thus, it is expected that the thermal stability of the PBSu/biochar nanocomposites would present an ameliorated thermal performance in comparison with the neat PBSu polyester. Overall, due to the highly promising character of the newly established PBSu/biochar nanocomposite materials based on their ameliorated mechanical performance and antibacterial properties, information about their thermal stability and degradation profile could also add scientific knowledge to the already established data. Thus, the thermal degradation profile of a newly synthesized material is a noteworthy priority; not only does it verify the required specifications of the product for a special application, but it also develops the range of future and possible applications.

**Figure 8:**
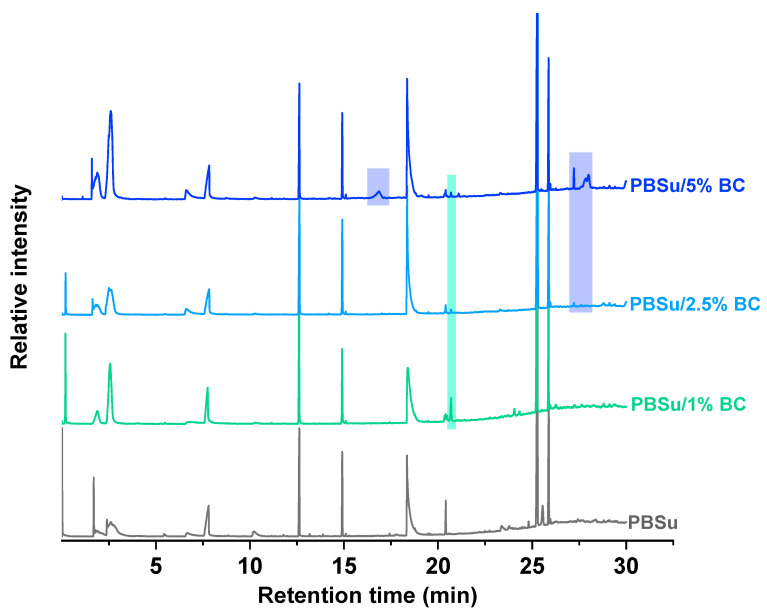
Total ion chromatograms (TICs) of PBSu prepared polyesters with different contents in biochar (1, 2.5, and 5 wt.%), pyrolyzed at 450 °C.

**Figure 9:**
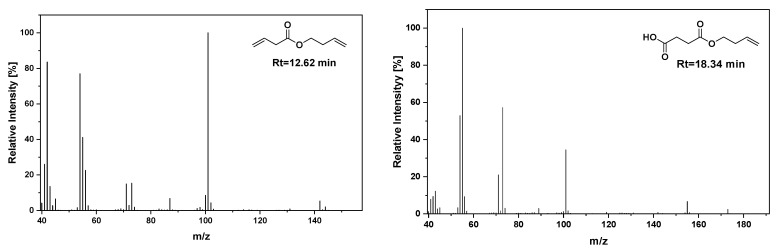

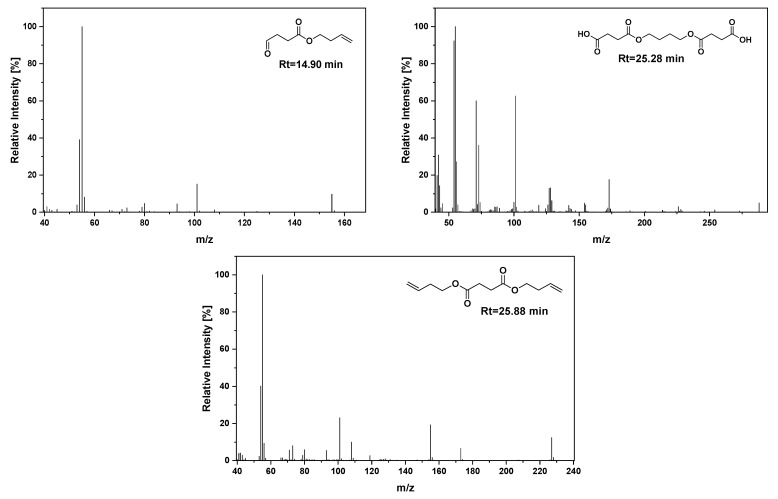
Selected m/z spectra of the characteristic pyrolysis products for neat PBSu.

**Figure 10:**
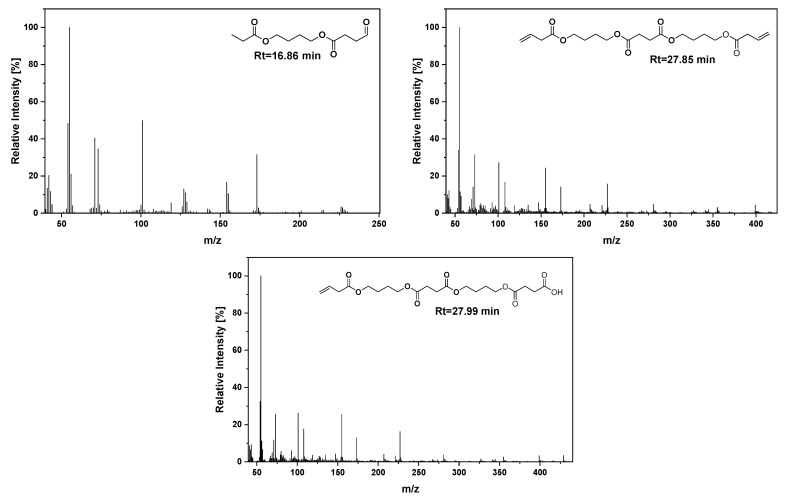
Mass spectra of newly detected pyrolysis products for the sample PBSu/biochar 5 wt.%.

**Scheme 1:**
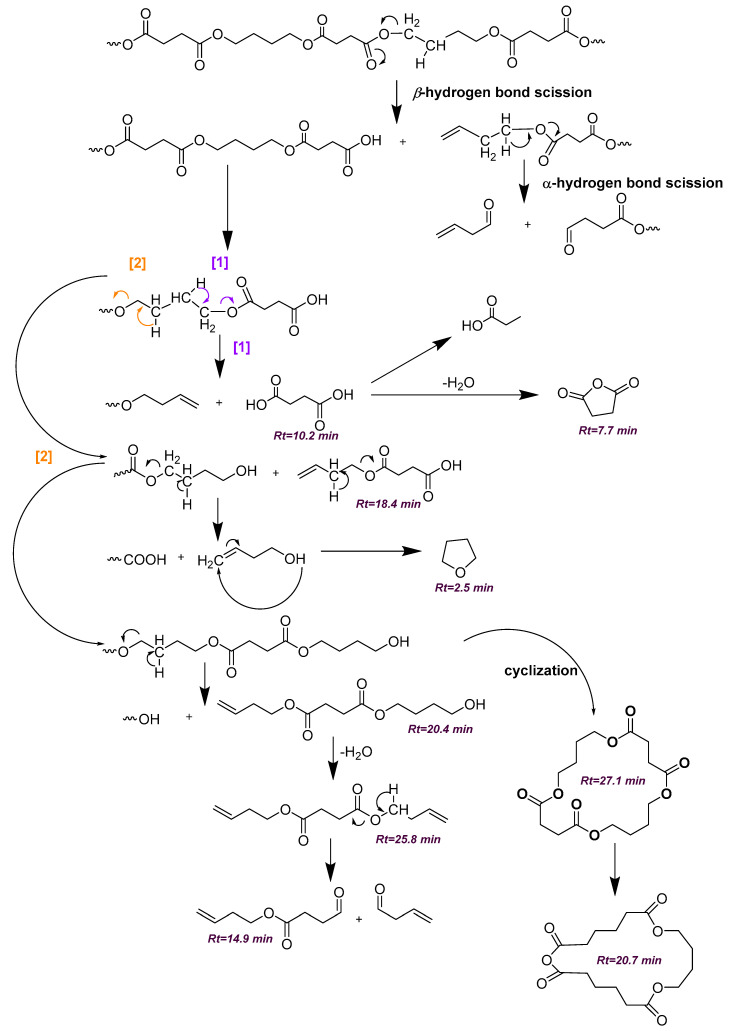
Proposed decomposition mechanism of PBSu.

In light of the above, flash pyrolysis (single shot) of PBSu and its nanocomposites with biochar in different concentrations was carried out at 450 °C, a temperature corresponding to that at which the majority of decomposition products are produced. This point is located in the middle of the initialization and at the end of the nanocomposite polyester decomposition procedure, as assumed from the TGA thermograms presented in Figure 5a. The pyrolysis total ion chromatographs (TICs) of the formed gas compounds for the four studied samples are depicted in Figure 8. The main peaks attributed to the principal degradation products were identified by mass spectrometry so as to construct the degradation pathway for the investigated samples (Figure 9). The mass spectra of the main pyrolysates at each retention time produced at 450 °C are presented in Figure 10. Subsequently, the identified pyrolytic products of PBSu nanocomposite materials are presented in Table 6, while the relative intensity of the possibly identified peaks are also included. The first pyrolysis products of neat PBSu included carbon dioxide at Rt = 1.69 min, tetrahydrofuran at Rt = 2.39 min, succinic anhydride at Rt = 7.76, and succinic acid at 10.19 min, respectively; in contrast, at higher retention times, allyl and diallyl compounds were dominant. These findings are in line with previous pyrolysis and PBSu thermal degradation studies [48,50]. The decomposition mechanism of PBSu mainly took place through β-hydrogen scission or β-decomposition of the polymeric backbone, which was characterized by a six-membered cyclic transition state. Concerning the hydroxyl-terminated compounds, these can be formed either via acyl-oxygen (C–O) homolytic scission or via hydrolysis of the β-decomposition products. For neat PBSu, among the degradation products, only one aldehyde was detected at Rt = 14.90 min, which was attributed to the homolytic α-hydrogen scission pathway. A proposed thermal decomposition pathway for neat PBSu is depicted in Scheme 1. According to the above, it could be concluded that the main degradation pathway for PBSu is β-hydrogen scission reaction. These findings align with the decomposition kinetics of neat PBSu. In detail, at the initial phase, the thermal degradation of neat PBSu was examined using TG measurements (Figure 6), revealing lower values (129 kJ·mol^−1^). This degradation process yielded carbon dioxide, tetrahydrofuran, succinic anhydride, and succinic acid products. As these byproducts were consumed, the degradation shifted toward the degradation of the β-hydrogen scission reaction, causing an increase in the activation energy of PBSu (146 kJ·mol^−1^).

According to Figure 8, the chromatograms of PBSu with 1, 2.5, and 5 wt.% biochar revealed, according to an initial observation, only slight differences provoked by the presence of the filler, since the TICs were almost identical. Actually, due to the high carbonized character of the used *Miscanthus* biochar (H/C < 0.3, VM (volatile matter) < 16%), the pyrolysis products displayed heavily discernible peaks [53]. The evolved products were identical, being detected with a similar pattern. However, some localized differences in the intensity of the peaks of some specific thermal degradation products can be recognized. In particular, for the PBSu/biochar 5 wt.% nanocomposite, new peaks at Rt = 16.8, 27.2, 27.8, and 27.9 min could be detected, implying a slight alteration of the thermal degradation of PBSu with the presence of biochar filler.

Despite the relatively low intensity of the new peaks, they are interesting for further analysis, since proof is provided regarding the effect of biochar on the mechanism of PBSu thermal degradation. For instance, a new peak at Rt = 16.86 min was displayed in the chromatogram of PBSu/biochar 5 wt.%, assigned to the formation of 4-(propionyloxy)butyl 4-oxobutanoate. Additionally, two peaks of small intensity could be distinguished in the chromatogram of the same sample at Rt = 27.22 and 28.02 min, attributed to 1,6,11,16-tetraoxacycloicosane-2,5,12,15-tetraone and bis(4-((4-oxobutanoyl)oxy)butyl) succinate, respectively. The narrow peak at Rt = 27.22 min was also detected for the sample PBSu/biochar 2.5 wt.%, but with lower intensity. One new peak attributed to but-3-en-1-yl (4-hydroxybutyl) succinate also appeared at Rt = 20.71 min, with a larger intensity for the nanocomposite with a lower biochar content. Interestingly, the peak at Rt = 25.54 min for neat PBSu attributed to the formation of 4-(4-(but-3-enoyloxy)butoxy)-4-oxobutanoic acid or butane-1,4-diyl bis(4-oxobutanoate) disappeared after the incorporation of the filler.

**Scheme 2:**
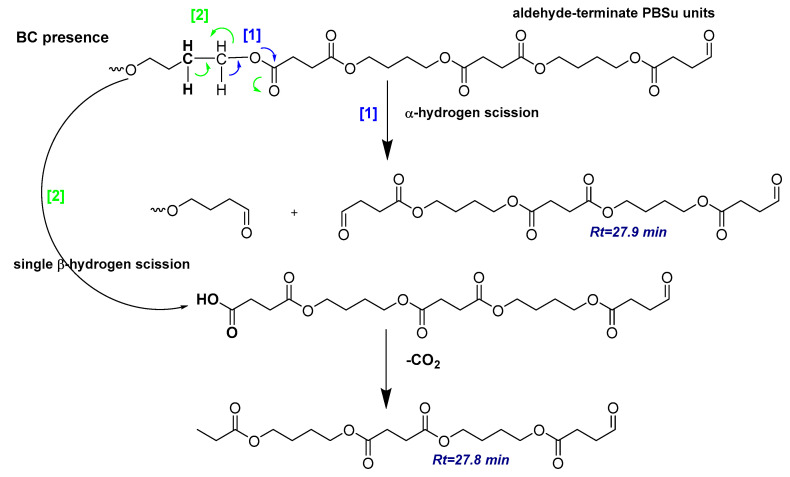
Possible degradation mechanism for the pyrolysis products produced in the presence of 5 wt.% of biochar filler.

Among the PBSu/biochar nanocomposites, at Rt < 10.2 min, the intensity of peaks was interestingly reduced for the PBSu/biochar 2.5 wt.% and subsequently increased for the sample PBSu/biochar 5 wt.%. This fluctuating trend was reversed for Rt > 12.62 min. From these data, it could be concluded for the sample PBSu/biochar 2.5 wt.% that the incorporation of the biochar filler catalyzed the formation of higher-molecular-weight compounds, while the formation of lower-molecular-weight compounds was more restricted. Moreover, it should be also noted that, for the peak found at Rt = 10.2 min, the intensity was reduced after the addition of the biochar, indicating that the filler hindered the evolution of succinic acid. A schematic illustration of the thermal degradation of PBSu nanocomposites after the incorporation of biochar is depicted in Scheme 2.

Overall, it can be concluded the neat PBSu and the PBSu/biochar nanocomposites presented a similar thermal degradation pathway for pyrolysis at 450 °C. The dominant decomposition mechanism seemed to be *β*-hydrogen scission and, to a lower extent, *α*-hydrogen scission, since the majority of the detected pyrolysis products included vinyl- and carboxylic acid-terminated compounds, with a lower content of aldehyde- and hydroxyl-terminated products. During the last minutes of retention (Rt > 16 min), some differences in the evolution profiles, especially for the nanocomposite with a higher content of filler, were detected, with the formation of some new thermal degradation products, possibly formed through the α-hydrogen bond scission path due to the catalyzing effect of biochar filler. This also implies that only some small modifications of the thermal degradation profile of the nanocomposites occurred in comparison with neat PBSu. These findings further support the decomposition kinetics. The thermal degradation of the PBSu/biochar 5 wt.% nanocomposite required less activation energy compared to neat PBSu. Furthermore, when the E values were calculated, the pre-exponential part of the PBSu/biochar 5 wt.% nanocomposite exhibited lower values in both stages compared to neat PBSu. As a result, the rate constant of the PBSu/biochar 5 wt.% mixture was significantly higher than that of pure PBSu, leading to an accelerated thermal degradation process. Hence, a minor modification, mainly related to the final decomposition steps for the prepared composite, could be justified.

## 3. Materials and Methods

### 3.1. Materials

Succinic acid (SA) (purum 99+%) and titanium isopropoxide (≥97%) (Tis) catalyst of analytical grade were purchased from Sigma-Aldrich Chemical Co (Saint Louis, MO, USA). 1,4-Butanediol (BD) (Purity: >99%) was obtained from Alfa Aesar (Kandel, Germany). Biobased biochar was synthesized from Miscanthus straw pellets using the Stage III pilot-scale pyrolysis unit at 700 °C [54]. It was provided by UK Biochar Research Center. It was dried overnight in an oven at 80 °C under vacuum conditions before each use. All other reagents were of analytical grade.

### 3.2. Nanocomposite Preparation

Nanocomposites of PBSu with biochar were prepared via the two-stage melt polycondensation method (esterification and polycondensation) using SA and BD in a molar ratio 1/1.1 and biochar at a concentration of 1, 2.5, and 5 wt.%, which was added to a 250 mL glass batch reactor simultaneously with SA and BD [55,56,57,58]. The reaction mixture was heated at 170 °C under nitrogen flow for 1 h and stirring speed 500 rpm, at 180 °C for an additional 1 h, and finally at 190 °C for 1.5 h. The first step of esterification was considered complete after the collection of the distilled amount of water in a graduated cylinder. In the stage of polycondensation (second step), the vacuum was gradually increased to 5.0 Pa over a period of about 30 min, to remove the excess diol or remaining H_2_O, to avoid excessive foaming, and to minimize oligomer sublimation. During this period, the temperature was gradually increased to 230 °C, while the stirring speed was also increased to 720 rpm. Then, 400 ppm of Tis catalyst was added to the reactor at the end of this stage. At this temperature, the reaction was kept constant for 30 min, and then increased by 10 °C every 30 min up to 250 °C, at which point the reaction was continued for 1.5 h (total polycondensation time 2.5 h). As the polycondensation reaction was completed, the polyesters were easily removed from the flask.

#### 3.2.1. X-ray Photoelectron Spectroscopy (XPS)

Kratos Analytical (Shimadzu Group Company, Manchester, UK) performed the X-ray photoelectron spectroscopy (XPS) of PBSu nanocomposites in film form. An Al–Ka1 X-ray source (energy 1486.6 eV) was used to generate XPS spectra, with a pass energy of 160 eV for survey scans and 20 eV for high-resolution spectra. The binding energies were calibrated using the binding energy (BE) of the C 1s peak, which was 284.6 eV ± 0.2 eV. The spectra were decomposed using the software’s least-squares fitting with a Gauss/Lorentz function and a Shirley background. Atomic ratios were calculated from background-subtracted peak areas using sensitivity factors provided by the data analysis system.

#### 3.2.2. Thermogravimetric Analysis (TGA)

Thermogravimetric analysis (TGA) was conducted on PBSu nanocomposites using a SETARAM SETSYS TG-DTA 16/18 instrument. The samples (6 ± 0.5 mg) were kept in crucibles made of alumina, and an empty one was used as a reference. To account for the effect of buoyancy, a blank measurement was taken and then subtracted from the experimental curve. PBSu nanocomposites were heated at rates of 5, 10, 15, and 20 °C/min while being maintained in a temperature range of 25–600 °C for the kinetic analyses [43]. The sample temperature, mass, first derivative, and heat flow were all continuously measured. The thermal degradation kinetic study of the PBSu nanocomposites was carried out using the NETZSCH Kinetics Neo program (NETZSCH, Selb, Germany) [59]. The goal of this model-fitting kinetic technique is to find a kinetic model that accurately describes the kinetics of the whole degradation reaction and has a small number of parameters that can be changed.

#### 3.2.3. Pyrolysis–Gas Chromatography/Mass Spectrometry Analysis (Py–GC/MS)

For Py–GC/MS analysis of the PBSu/biochar nanocomposites, a very small amount of each material was “dropped” initially into the “double-shot” EGA/PY (evolved gas analysis/pyrolysis) 3030D Pyrolyzer (Frontier Laboratories Ltd., Fukushima, Japan) using a CGS-1050Ex carrier gas selector. For pyrolysis analysis (flash pyrolysis), each sample was placed into the sample cup, which subsequently fell into the Pyrolyzer furnace. The pre-selected pyrolysis temperature was set at 450 °C, while the GC oven temperature was programmed at 50 °C for 2 min, followed by a stepped increase to 200 °C with a heating rate of 5 °C/min, where it was held for 8 min; then, the temperature was increased at 300 °C by a rate of 20 °C/min, where it was held for 5 min. Sample vapors generated in the furnace were split at a ratio of 1/50; then, a portion moved to the column at a flow rate of 1 mL/min and pressure of 53.6 kPa, and the remaining portion exited the system via the vent. The pyrolysates were separated using a temperature-programmed capillary column in a Shimadzu QP-2010 Ultra Plus (Kyoto, Japan) gas chromatogram and analyzed by the mass spectrometer MS-QP2010SE of Shimadzu (Kyoto, Japan). The Ultra-ALLOY^®^ metal capillary column from Frontier Laboratories LTD (Fukushima, Japan) was used containing a 5% diphenyl and 95% dimethylpolysiloxane stationary phase, with a column length of 30 m and column ID of 0.25 mm. For the mass spectrometer, the following conditions were used: ion source heater 200 °C, interface temperature 300 °C, vacuum 10^−4^–10^0^ Pa, m/z range 40–500 amu (atomic mass unit), and scan speed 10,000. The ion gas chromatograms and spectra retrieved by each experiment were subjected to further interpretation through Shimadzu (NIST11.0) and Frontier post-run software (F-Search software 4.3). Identification was confirmed depending on the similarity percentage (minimum value of 80%) across the average mass spectra of the entire chromatogram.

## 4. Conclusions

Global environmental concerns have accelerated worldwide efforts for the development of alternative materials that are biobased and fully biodegradable in order to replace nondegradable, fossil-based polymers in the near future. In this study, the thermal stability and decomposition mechanism of PBSu/biochar 1, 2.5, 5 wt.% nanocomposites were thoroughly investigated. XPS measurements of pristine PBSu and its nanocomposite containing 5 wt.% biochar revealed that biochar addition resulted in a slightly higher binding energy of O 1s and C 1s due to the slightly higher carbon content on the surface. The TGA curves depicted that the biochar has a potential catalytic impact because of the slight decrease in thermal stability. Furthermore, the residue content of the PBSu nanocomposites at 600 °C was found to be increased upon increasing the biochar content, due to the accumulation of a higher char amount able to persist at high temperatures. The study of degradation kinetics through thermogravimetry showed that the thermal decomposition of the PBSu/biochar 5 wt.% nanocomposite required less activation energy than that of neat PBSu. Concerning the investigation of the thermal degradation mechanism through Py–GC/MS analysis, it was proven that neat PBSu and PBSu/biochar nanocomposites presented a similar thermal degradation pathway for pyrolysis at 450 °C. The main decomposition mechanism seemed to be β-hydrogen scission. This is because the majority of the detected pyrolysis products included vinyl- and carboxylic acid-terminated compounds. Only for the nanocomposite with a higher biochar content was the formation of some new thermal degradation products observed in the last few minutes of retention, which were possibly formed through the α-hydrogen bond scission path. This could be attributed to the catalytic impact of biochar filler when added at a higher content. This work provides further information for the application of PBSu/biochar nanocomposites as agricultural mulch films or in printing electronics. In light of the above, future work could investigate the aging effects of the bionanocomposites produced in the framework of this study.

## Figures and Tables

**Table 1:** Area percentage of biochar determined from the XPS spectrum of C 1s.

Components	Binding Energy (BE)	Area (%)
Carbon sp^2^ (C=C)	284.0	29.7
Carbon sp^3^ (C–C)	284.6	32.7
C–O	285.7	19.3
Carbonyl (C=O)	287.4	2.2
O=C–OH	288.7	4.6
K2p3/2	292.9	7.6
K2p1/2	295.6	4.0

**Table 2:** Area percentage of neat PBSu and PBSu/biochar 5 wt.% nanocomposites determined from the XPS spectrum of C 1s.

Neat PBSu			PBSu/Biochar 5 wt.%		
Components	Binding Energy (BE)	Area (%)	Components	Binding Energy (BE)	Area (%)
Carbon sp^2^ (C=C)	283.9	8.7	Carbon sp^2^ (C=C)	283.9	9.6
Carbon sp^3^ (C–C)	284.6	24.9	Carbon sp^3^ (C–C)	284.6	19.7
C*–NH_2_, C*–C=O	285.8	10.9	C*–NH_2_, C*–C=O	285.8	34.8
C*–O	286.9	17.2	C–O	286.9	19.1
C*=O	288.2	27.4	C=O	288.3	7.9
C*–OH	289.6	4.3	C-OH	289.5	5.0
O–C*=O	290.5	6.6	O–C*=O	290.1	3.9

**Table 3:** Deconvoluted peak assignments for high-resolution XPS scan of O 1s and N 1s regions of PBSu/biochar 5 wt.% nanocomposite.

**A/A**	C=O*		C–O*		O–C=O*	
	**BE** **(eV)**	**Area** **(%)**	**BE** **(eV)**	**Area** **(%)**	**BE (eV)**	**Area** **(%)**
PBSu	531.3	29.3	532.8	47.8	534.7	22.9
PBSu/5% Biochar	531.5	33.1	533.0	50.1	534.5	16.8
**A/A**	C–N*		N*–H			
	**BE** **(eV)**	**Area** **(%)**	**BE** **(eV)**		**Area** **(%)**	
PBSu	399.3	47.7	400.2		52.3	
PBSu/5% Biochar	399.4	50.5	400.8		49.5	

**Table 4:** TGA results of all studied samples.

Sample	T0.5 (°C)	T2.5 (°C)	T5 (°C)	Td, Max (°C)
Neat PBSu	336.1	376.9	389.9	443.3
PBSu/biochar 1 wt.%	333.3	365.3	381.3	439.4
PBSu/biochar 2.5 wt.%	332.2	368.6	381.0	431.9
PBSu/biochar 5 wt.%	333.1	368.2	380.7	429.7

**Table 5:** Activation energy, pre-exponential factor, and reaction order of neat PBSu and PΒSu/biochar 5 wt.% nanocomposite.

Sample	Model	Activation Energy, E (kJ·mol^−1^)	Pre-Exponential Factor, logA_1_ (s^−1^)	Reaction Order (n)	Log K_cat_	Contribution	R^2^
Neat PΒSu	Cn	122.94	7.068	0.493	0.569	0.237	0.99997
	Cn	151.38	8.686	1.454	1.181	0.763	
PBSu/biochar 5 wt.%	Cn	117.27	6.679	0.368	0.744	0.249	0.99990
	Cn	138.50	7.935	1.339	1.113	0.752	

**Table 6:** Thermal decomposition products of neat PBSu and PBSu/biochar nanocomposites.

Retention Time (min)	Sample Name				M_w_ (amu)	Assigned Compound
	PBSu Neat	PBSu/BC 1 wt.%	PBSu/BC 2.5 wt.%	PBSu/BC 5 wt.%		
	Relative Intensity (%)					
1.8	19.73	4.60	3.51	5.12	44	CO, CO_2_
2.5	7.59	20.25	8.51	19.71	42, 54, 72	Propionic acid 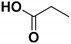 or Tetrahydrofuran 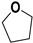
7.7	11.12	12.27	8.62	11.49	42, 56, 100	Succinic anhydride 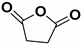
10.2	2.75	0.64	0.55	0.86	43, 57, 75, 87, 100, 118	Succinic acid 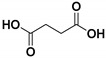
12.6	36.06	37.79	41.60	38.64	45, 54, 73, 87, 101, 114, 131, 142	But-3-en-1-yl but-3-enoate 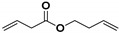
14.90	28.03	25.09	31.72	28.91	45, 55, 73, 80, 101, 108, 125, 155	But-3-en-1-yl 4-oxobutanoate 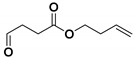
18.4	27.00	18.82	42.88	40.2	43, 55, 73, 89, 101, 119, 155, 174	4-(but-3-en-1-yloxy)-4-oxobutanoic acid 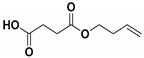
16.85	n.d.	n.d.	n.d.	4.17	55, 71, 101, 128, 154, 173, 201, 230	4-(propionyloxy)butyl 4-oxobutanoate 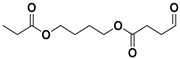
20.4	12.08	3.50	3.33	3.36	45, 55, 73, 89, 101, 119, 155, 213, 244	But-3-en-1-yl (4-hydroxybutyl) succinate 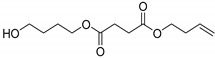
20.7	n.d.	10.2	2.9	6.1	55, 69, 81, 95, 109, 121, 136, 149, 175, 272	1,6,13-trioxacyclononadecane-7,12,14,19-tetraone 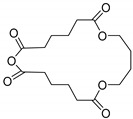
25.2	100.00	100.00	100.00	100.00	45, 55, 73, 80, 101, 108, 119, 155, 173, 227, 273, 288	4,4′-(butane-1,4-diylbis(oxy))bis(4-oxobutanoic acid) 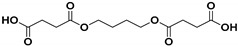
25.6	11.44	n.d.	n.d	n.d.	43, 57, 69, 83, 97, 111, 125, 147, 196, 224, 258	4-(4-(but-3-enoyloxy)butoxy)-4-oxobutanoic acid 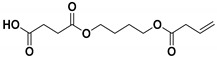 or Butane-1,4-diyl bis(4-oxobutanoate) 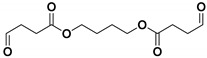
25.8	53.61	49.79	55.5	47.11	45, 55, 73, 80, 101, 108, 119, 155, 173, 228	Di(but-3-en-1-yl) succinate 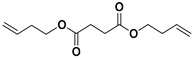
27.1	n.d.	n.d.	5.05	10.44	41, 55, 69, 81, 95, 109, 121, 136, 149, 175, 207, 281, 344	1,6,11,16-tetraoxacycloicosane-2,5,12,15-tetraone 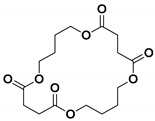 or 4-(4-((4-(but-3-en-1-yloxy)-4-oxobutanoyl)oxy)butoxy)-4-oxobutanoic acid 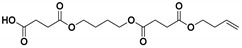
27.8	n.d.	n.d.	n.d.	6.80	55, 73, 101, 108, 155, 173, 227, 281, 355, 399	4-((4-oxobutanoyl)oxy)butyl (4-(propionyloxy)butyl) succinate 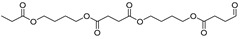
27.9	n.d.	n.d.	n.d.	8.67	55, 73, 101, 108, 155, 173, 227, 281, 355, 399, 429	bis(4-((4-oxobutanoyl)oxy)butyl) succinate 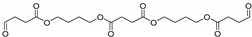

## Data Availability

All the data of this study are included in the manuscript.

## Sample Availability

Samples of the studied compounds are not available from the authors.
